# Stent-in-stent placement of multi-hole metal stents (M2) following creation of a gateway by endoscopic ultrasound-guided antegrade stenting after failed balloon assisted endoscopy

**DOI:** 10.1055/a-2791-4460

**Published:** 2026-02-13

**Authors:** Hirotsugu Maruyama, Kojiro Tanoue, Yuji Kawata, Tatsuya Kurokawa, Yoshinori Shimamoto, Yuki Ishikawa-Kakiya, Yasuhiro Fujiwara

**Affiliations:** 1 12935 Department of Gastroenterology, Graduate School of Medicine, Osaka Metropolitan University, Osaka, Japan

Endoscopic biliary drainage for malignant hilar biliary obstruction should ideally allow for stent removal, regardless of the stent type 1 . When balloon assisted endoscopy (BAE) fails, interventional endoscopic ultrasound (EUS) becomes an alternative; however, in surgically altered anatomy, EUS-guided hepaticoduodenostomy is not indicated, and right-sided drainage via the left intrahepatic route is extremely challenging – especially when the entire procedure must be completed in one session, often preventing the placement of removable stents.

In this case, because future chemotherapy was expected, stent removability was prioritized 2 3 . After unsuccessful BAE, EUS-guided antegrade stenting was used to create a gateway, allowing the successful transpapillary stent-in-stent placement of multi-hole self-expandable metal stents (MHSEMSs).

A 67-year-old woman who had undergone gastrojejunostomy for duodenal invasion caused by gallbladder cancer had two inside plastic stents (PSs) placed for obstructive jaundice. He was later admitted for the treatment of acute cholangitis. Because of the duodenal obstruction, reintervention was attempted using BAE through the bypass. Although the PSs were removed, biliary cannulation failed and drainage could not be achieved ( Fig. 1 ). Since the major papilla was reachable, we employed a combined interventional EUS and endoscopic retrograde cholangiopancreatography (ERCP) approach.

**Fig. 1:**
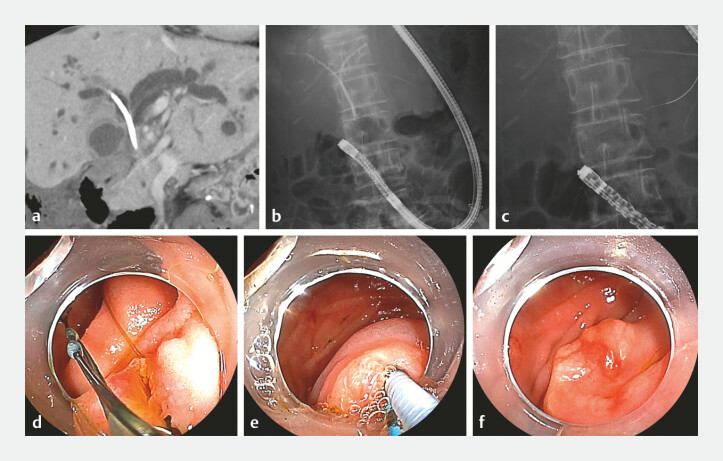
Preoperative computed tomography (CT) and balloon-assisted endoscopy (BAE) images. **a** Bilateral intrahepatic bile duct dilatation and pre-existing plastic stents were observed. **b** A fluoroscopic image during BAE showing two inside plastic stents. **c** A fluoroscopic image after the removal of the inside plastic stents showing a guidewire placed in the pancreatic duct. **d** An endoscopic image of the major papilla showing the retrieval suture of the inside plastic stents before their removal. **e** An endoscopic image showing an attempt at the pancreatic duct guidewire technique. **f** An endoscopic image taken at the moment when biliary cannulation was abandoned.

First, under EUS guidance, a MHSEMS was deployed antegradely to create a large transpapillary gateway that would allow easier access ( Fig. 2 ). Then, a colonoscope (PCF-H290ZI; Olympus, Tokyo, Japan) was advanced to the major papilla, and using the MHSEMS as an access route, a guidewire was inserted into the right intrahepatic duct. Another MHSEMS was subsequently deployed ( Video 1 , Fig. 3 ). The patient was discharged without any adverse events.

**Fig. 2:**
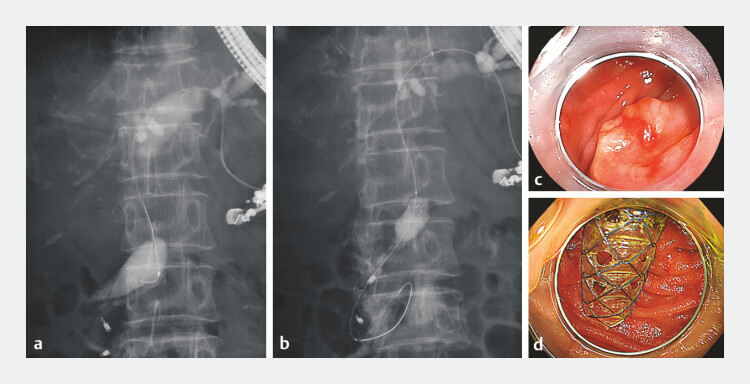
A fluoroscopic image of interventional endoscopic ultrasound, along with endoscopic images before and after creating the gateway. **a** Cholangiography after B3 puncture. A stenosis was identified in the hilar bile duct region. **b** A fluoroscopic image showing the placement of a multi-hole self-expandable metal stent (MHSEMS) from the left intrahepatic bile duct to the duodenum via the major papilla. **c** and **d** Endoscopic images before and after creating the gateway using the MHSEMS.

Stent-in-stent placement of multi-hole metal stents (M2) following creation of a gateway by endoscopic ultrasound-guided antegrade stenting.Video 1

**Fig. 3:**
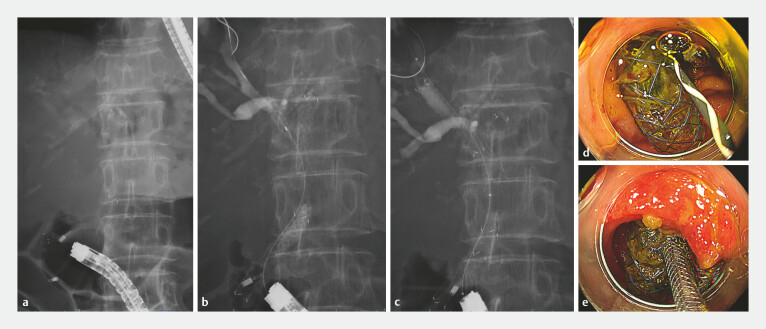
Endoscopic images of the endoscopic procedure performed with a colonoscope after creating the gateway. **a** A fluoroscopic image at the time of reaching the major papilla. **b** A fluoroscopic image showing biliary cannulation through the gateway and placement of a guidewire into the right intrahepatic bile duct through the hole of the multi-hole self-expandable metal stent (MHSEMS). **c** A fluoroscopic image showing the stent-in-stent placement using the MHSEMS. **d** An endoscopic image showing the guidewire placement into the bile duct through the gateway. **e** An endoscopic image showing the insertion of the second MHSEMS through the gateway.

This technique enables secure biliary access even in cases with poor endoscope maneuverability by creating a wide gateway and offers the advantages of removability and potentially prolonged stent patency. This approach represents a next-generation option that integrates the strengths of both EUS and ERCP.

Endoscopy_UCTN_Code_TTT_1AS_2AH

## References

[1] Kanno Y Ito K Nakahara K Suprapapillary placement of plastic versus metal stents for malignant biliary hilar obstructions: a multicenter, randomized trial Gastrointest Endosc 2023 98 211 221 10.1016/j.gie.2023.03.007 36907528

[2] Maruyama H Ishikawa-Kakiya Y Kawata Y Stent-in-stent deployment across the papilla for malignant hilar biliary obstruction using novel slim multi-hole metal stents Endosc Int Open 2025 13 a27142453 10.1055/a-2714-2453 PMC12551655 41142262

[3] Ogura T Uba Y Kanadani T Reintervention for recurrent biliary obstruction after stent-in-stent deployment of multi-hole self-expandable metal stents Endoscopy 2025 57 E181 E182 10.1055/a-2534-3143 39978392 PMC11842147

